# Experimental Study on the Effect of Hydroxyethyl Cellulose on the Friction-Reducing Performance of Thixotropic Slurries in Pipe Jacking Construction

**DOI:** 10.3390/ma18133155

**Published:** 2025-07-03

**Authors:** Xiao Yu, Yajun Cao, Fubing Tian, Chaowei Chen, Chao Chen, Wei Wang, Yaru Jiang

**Affiliations:** Key Laboratory of Ministry of Education for Geomechanics and Embankment Engineering, Hohai University, Nanjing 210024, China; yuxiao0401@outlook.com (X.Y.); hhuccw@163.com (C.C.); cchao@hhu.edu.cn (C.C.); wwang@hhu.edu.cn (W.W.);

**Keywords:** pipe jacking construction, thixotropic slurry mix design, orthogonal experiment, hydroxyethyl cellulose

## Abstract

In pipe jacking construction, thixotropic slurry critically governs lubrication, friction reduction, and ground support. This study evaluated slurry performance through six parameters: specific gravity (SG), pH, fluid loss (FL), water separation rate (WSR), filter cake thickness (FCT), and funnel viscosity (FV). Orthogonal experiments optimizing bentonite, carboxymethyl cellulose (CMC), and sodium carbonate (Na_2_CO_3_) ratios established 10 wt.% bentonite, 0.3 wt.% CMC, and 0.4 wt.% Na_2_CO_3_ as the optimal formulation. Subsequently, to address performance limitations in challenging conditions, this study introduces hydroxyethyl cellulose (HEC) as a novel additive, with potential advantages under high-salinity and variable pH conditions. Comparative experiments demonstrated that HEC, as a non-ionic water-soluble cellulose, not only significantly increases FV and reduces FL while maintaining SG, FCT, and WSR within acceptable thresholds, but also exhibits superior pH stability compared to CMC. Based on the aforementioned results, interface friction characterization tests were conducted on representative slurry formulations with varying FVs, quantitatively demonstrating the viscosity-dependent friction-reduction performance. Complementary scanning electron microscopy (SEM) analysis of three distinct thixotropic slurry compositions systematically revealed their microstructural characteristics, with microscopic evidence confirming the excellent compatibility between HEC and thixotropic slurry matrix. These findings highlight HEC’s potential as an effective alternative in pipe jacking, particularly in demanding geological environments.

## 1. Introduction

Pipe jacking technology, as an advanced trenchless technology for underground pipeline installation, has seen extensive application in urban utility networks and major infrastructure projects—including transport tunnels, hydraulic systems, and utility corridors—due to its minimal surface disruption and ecological benefits [1,2]. The technique operates by hydraulically or mechanically thrusting prefabricated pipe segments through the ground while excavating the tunnel face. Nevertheless, substantial technical challenges persist, particularly with interfacial friction between pipes and surrounding strata. As projects encounter longer distances, larger diameters [3,4,5,6,7,8], or complex geologies [9,10,11,12,13,14,15], frictional resistance exhibits nonlinear escalation. This not only demands higher jacking forces but also risks construction defects (e.g., pipe misalignment) and geohazards (e.g., ground settlement), especially in high-water-table or soft-soil conditions. In practical engineering, various engineering measures have been adopted to reduce the jacking force, such as grouting with thixotropic slurry and installation of intermediate jacking stations [16,17].

The thixotropic slurry friction-reduction technique addresses this challenge by forming interfacial lubricating films. Injection of the slurry into the pipe-strata annular gap establishes a stable lubricating layer that significantly reduces friction coefficients and enhances jacking performance—with formulation quality being the primary determinant of effectiveness [18,19,20,21,22]. Conventional thixotropic slurries typically contain bentonite, carboxymethyl cellulose (CMC), sodium carbonate (Na_2_CO_3_), and water. As the key thickening agent, CMC enables stable lubricating film formation through its combined shear-thinning behavior and water-retention capabilities.

Recent research on thixotropic slurry technology for complex geological applications has evolved along two parallel tracks: performance enhancement through multicomponent additives and the development of environmentally sustainable formulations. Microbial-induced calcium carbonate precipitation (MICP) technology has emerged as a particularly promising approach, demonstrating the ability to simultaneously reduce interfacial friction coefficients and reinforce adjacent strata through bacterially mediated calcification processes [23]. In parallel, boronic acid-crosslinked polymer systems have been successfully incorporated into polyacrylamide-bentonite slurries, effectively addressing the biodegradation limitations characteristic of conventional materials [24]. The strategic combination of plant-derived additives (gums and potassium humate) with synthetic components (sodium carboxymethyl cellulose and graphite powder) has yielded measurable improvements in overall slurry performance [8], while nanomaterial additives have shown particular efficacy in enhancing both thixotropic behavior and rheological properties [17]. Furthermore, the technological synergy with drilling fluid systems, which share fundamental compositional and functional characteristics with thixotropic slurries, has generated several significant innovations, including core-shell nano acrylic resin/SiO_2_ composites for enhanced wellbore stability in shale gas applications [25], laponite nanoparticles for improved thermal stability [26], and specialized cellulose derivatives for precise rheological modification [27].

In addition, cellulose ether modification research [28,29,30,31] offers new avenues for slurry enhancement. Hydroxyethyl cellulose (HEC), sharing water-soluble cellulose ether characteristics with CMC, demonstrates superior thickening, water retention, and rheological performance [27,32,33,34,35,36,37], with established applications in food [38], pharmaceuticals [39,40], 3D printing, and oilfields. Compared to CMC, HEC’s non-ionic nature confers enhanced acid stability and salt tolerance, particularly advantageous in pH-fluctuating or high-salinity strata. These attributes position HEC as a promising candidate for thixotropic slurry optimization in geologically challenging environments.

While HEC has demonstrated efficacy in conventional drilling fluids, its performance in pipe jacking slurry applications remains insufficiently characterized. To investigate the effects of different additives on the performance of thixotropic slurries and assess the feasibility of applying HEC in thixotropic slurries, this study first employed orthogonal experimental design to systematically investigate bentonite-CMC-Na_2_CO_3_ formulations, quantitatively determining the hierarchical influence of constituent components. Subsequently, through the innovative incorporation of HEC as a novel thickener, comparative experiments revealed HEC’s exceptional capability in enhancing thixotropic slurry performance metrics. To visually demonstrate the viscosity-dependent friction-reduction performance of the slurries, interface friction characterization tests were conducted on representative formulations with varying funnel viscosities. Building on these macroscopic findings, microstructural characterization via scanning electron microscopy (SEM) was conducted on three distinct formulations—bentonite-CMC-Na_2_CO_3_, bentonite-CMC, and bentonite-HEC—to analyze their structural characteristics. This paper establishes a new technical pathway for slurry material optimization, offering both theoretical significance and engineering application potential.

## 2. Materials and Methods

### 2.1. Materials and Slurry Preparation

**Figure 1:**
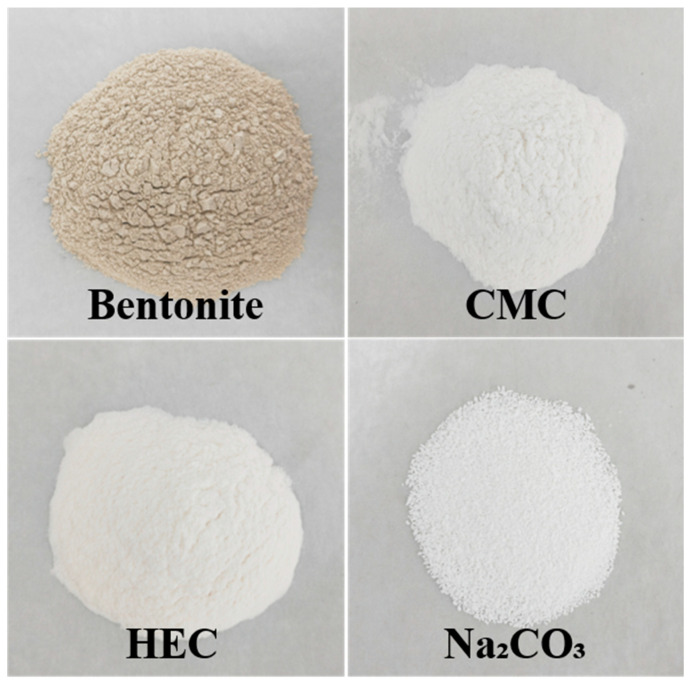
Raw materials.

Figure 1 displays the four materials used in thixotropic slurry, whose complete specifications are provided in Table 1.

This study established standardized preparation procedures for the following two distinct formulation systems.

For the Na_2_CO_3_-containing system, preparation commenced with precise weighing of components according to formulated ratios. The standardized protocol consisted of four consecutive stages: (i) 2 min dry blending of bentonite and CMC, (ii) incremental addition of 5% Na_2_CO_3_ solution in three aliquots with continuous 3 min mixing, (iii) incorporation of remaining water accompanied by 5 min wet mixing, and (iv) final 24 h temperature-controlled hydration in sealed containers.

For the Na_2_CO_3_-free system, preparation commenced with precision weighing of bentonite and selected cellulose (CMC or HEC), progressing through sequential phases: (i) initial 2 min dry mixing of bentonite with selected cellulose (CMC or HEC), (ii) transfer to mixing tank and subsequent 8 min aqueous mixing for complete dispersion, (iii) application of the identical temperature-controlled curing protocol as the Na_2_CO_3_ system, and (iv) concluding 24 h sealed hydration phase.

All specimens were prepared in triplicate (n = 3). Experimental data underwent outlier removal via Grubbs’ criterion prior to calculating arithmetic means as final results.

### 2.2. Experimental Methods

This study developed a comprehensive performance evaluation system based on six critical parameters: specific gravity (SG), marsh funnel viscosity (FV), pH, fluid loss (FL), filter cake thickness (FCT), and water separation rate (WRS). Table 2 presents the control requirements and experimental instruments for the six performance criteria. The detailed control principles are as follows: (i) Inadequate SG may result in insufficient formation support, whereas excessive SG can significantly impair fluid mobility; (ii) An optimal FV range (100–120 s) ensures favorable fluidity to effectively fill voids while preventing excessive infiltration-induced ground deformation. Geological heterogeneity necessitates site-specific viscosity requirements, and this study employs this interval as a critical criterion for superior slurry selection; (iii) Alkaline conditions (pH > 11) induce colloidal agglomeration and phase separation, while acidic environments (pH < 8) substantially accelerate corrosion rates of metallic conduits; (iv) To comply with anti-seepage specifications, the FL under 0.69 MPa pressure must not exceed 25 mL over a 30 min filtration period [41]; (v) FCT exhibits an operational trade-off: insufficient thickness raises leakage risks, while excessive accumulation increases frictional resistance at pipe–wall interfaces, demanding precision during slurry deployment; (vi) Engineering protocols strictly mandate thixotropic slurries to exhibit zero WSR throughout a 24 h static settlement, ensuring sustained homogeneity and suspension stability during construction processes [41].

**Figure 2:**
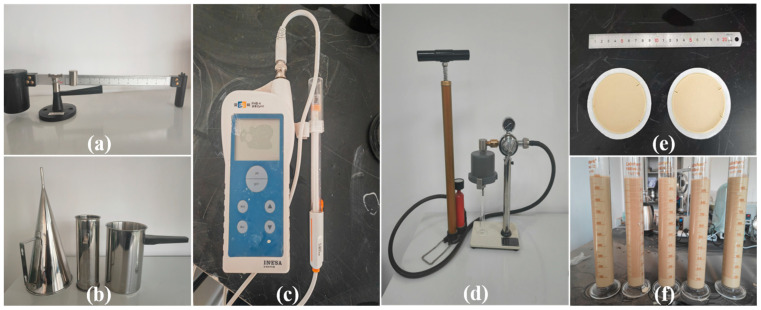
Experimental instruments: (**a**) NB-1 mud balance, (**b**) marsh funnel, (**c**) PHB-4 portable pH meter, (**d**) ZNA-2A filter press, (**e**) filter cake and caliper, (**f**) 1000 mL graduated cylinder.

Figure 2 illustrates experimental instruments utilized in the experimental procedures.

### 2.3. Experimental Design

#### 2.3.1. Orthogonal Experimental Design and Range Analysis

Orthogonal experimental design is an efficient, rapid, and economical methodology for investigating multi-factor and multi-level systems. When experiments involve numerous factors and potential interactions between them, this approach addresses challenges such as excessive experimental workloads and impractical implementation, achieving results equivalent to comprehensive full-factorial trials with significantly fewer experimental runs. The methodology typically employs predefined orthogonal arrays to structure experiments. Upon data collection, the optimal combination of factor levels and the relative influence strength of each factor are determined through range analysis or analysis of variance, thereby fulfilling experimental objectives. Currently, orthogonal experimental design has been widely adopted across diverse fields, including materials science, chemical engineering, pharmaceutical development, and industrial manufacturing.

In this study, an orthogonal design was employed to examine the synergistic effects of sodium bentonite content, CMC content, and Na_2_CO_3_ content on slurry performance. Based on the multi-factor, multi-level orthogonal design principle, a three-factor, four-level orthogonal array (L_16_(4^3^)) was implemented to systematically analyze the interaction mechanisms among these components [17]. The experimental factor-level configuration is presented in Table 3.

The experimental results were analyzed using the range analysis method [42]. Range analysis determines the influence intensity of factors by calculating average values (K) of the corresponding indicators at different levels of each factor. The range value (R = K_max_ – _min_) is used to assess the significance of each factor: a larger range indicates a more significant influence of the factor on the performance indicator. The ranking of range values determines the primary and secondary relationships among factors, guiding the optimization direction of the formulation.

#### 2.3.2. Comparative Experimental Design

Comparative experiments are designed based on the single-variable control principle, a systematic research methodology that strictly limits experimental conditions to adjust only the target independent variable (e.g., type and dosage of thickening agents) while maintaining other foundational variables constant (e.g., bentonite content). This approach eliminates interference from non-research factors and enables precise elucidation of the impact mechanisms of the target variables on system performance.

Building upon orthogonal experimental investigation of CMC’s influence on thixotropic slurry performance, this study established a binary control system to systematically evaluate the feasibility of substituting CMC with HEC as the thickening agent. The experimental design incorporated parallel formulations: an experimental group combining 8 wt.% sodium bentonite with HEC (0.2–1.0 wt.%) and a control group maintaining 8 wt.% sodium bentonite with CMC (0.2–1.0 wt.%) for direct performance comparison.

The detailed mix proportion parameters for each group are presented in Table 4. Through systematic comparative analysis of the effects of HEC and CMC on key slurry performance indicators—including SG, pH, FL, WSR, FCT, and FV—this study evaluates the performance enhancement effects of HEC across varying dosage gradients (0.2–1.0 wt.%) relative to CMC. This analysis aims to demonstrate the potential application of HEC in thixotropic slurry systems.

**Figure 3:**
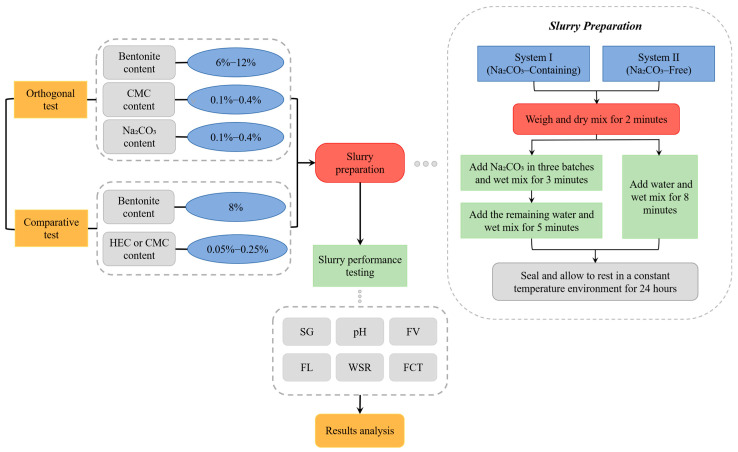
Schematic of experimental procedure and slurry formulation.

Figure 3 details the workflow of orthogonal experiments and comparative experiments, along with the slurry preparation procedures.

### 2.4. Interfacial Friction Property Testing Experiment

Engineering practice demonstrates that the friction-reduction capability of slurries is predominantly governed by their viscous properties. After screening several representative viscosity groups through orthogonal experiments and comparative experiments, a series of slurry–stratum interface friction characteristic tests were conducted to visually demonstrate the differences in friction-reduction efficiency among slurries with varying FVs.

**Figure 4:**
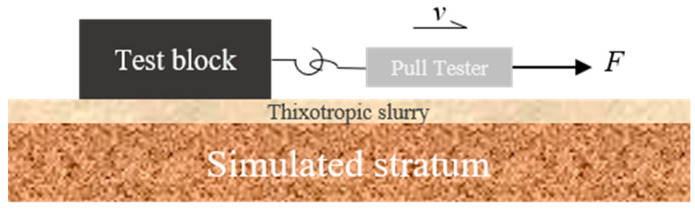
Schematic diagram of the slurry–stratum interface friction test model.

**Figure 5:**
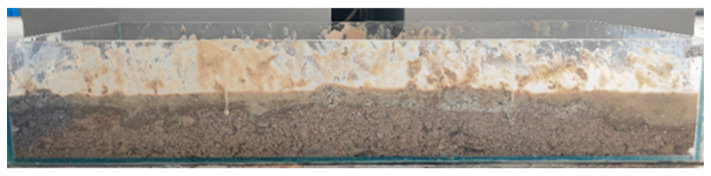
Side view of the slurry–stratum structure.

The experiment utilized test blocks with identical bottom surface roughness and geometric dimensions (mass set at 1, 1.5, and 2 units), which were placed on the surface of a pre-established slurry–stratum model, as shown in Figure 4, the schematic diagram of the slurry–stratum interface friction test model, and Figure 5, the side view of the slurry–stratum structure. A pull tester was employed to pull the test blocks at a constant speed. Once the test data stabilized, readings were recorded, with ten sets of valid data collected for each mass gradient. The resulting dataset enabled generation of friction force versus normal stress scatter plots, from which interfacial friction coefficients and adhesion forces were derived via linear regression analysis.

### 2.5. SEM Microstructural Observation

**Figure 6:**
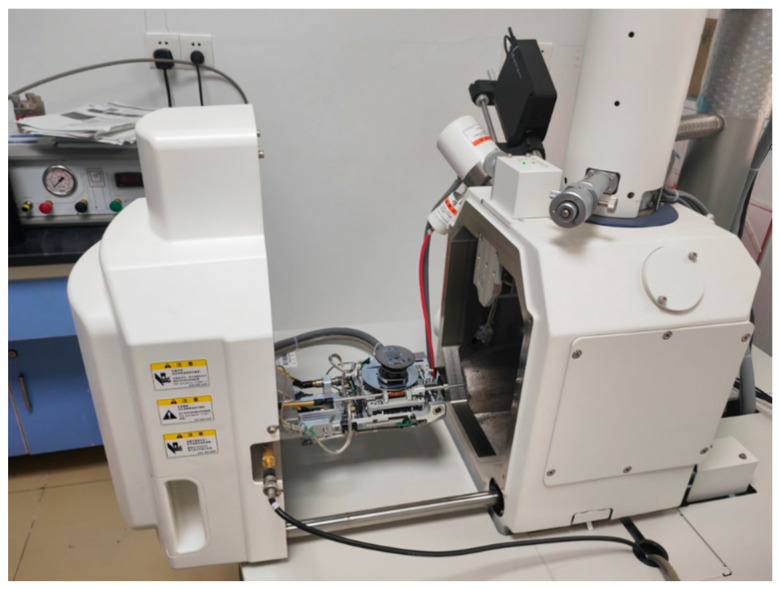
Hitachi SU3500 field-emission SEM system.

The microstructural characteristics of three slurry systems—bentonite-CMC-Na_2_CO_3_, bentonite-CMC, and bentonite-HEC—were systematically investigated using a Hitachi SU3500 (Hitachi High-Technologies Corporation, Tokyo, Japan) field-emission scanning electron microscope (Figure 6). Sample preparation involved oven-drying at 55 °C for 6 h to constant weight, followed by ion-sputter gold coating to ensure adequate conductivity. High-resolution imaging was conducted in secondary electron imaging (SEI) mode at progressively increasing magnifications of 400× for macrostructural evaluation, 1500× for interfacial characterization, and 3000× for microstructural examination.

## 3. Results and Discussion

### 3.1. Analysis of Orthogonal Experimental Results

The orthogonal experimental results are presented in Table 5. Based on the experimental data, the mean (K) and range (R) for each influencing factor were calculated to quantify their impacts on thixotropic slurry performance. Through range analysis, the factors were then ranked by significance, and the optimal combination within the three-factor concentration range was determined.

**Figure 7:**
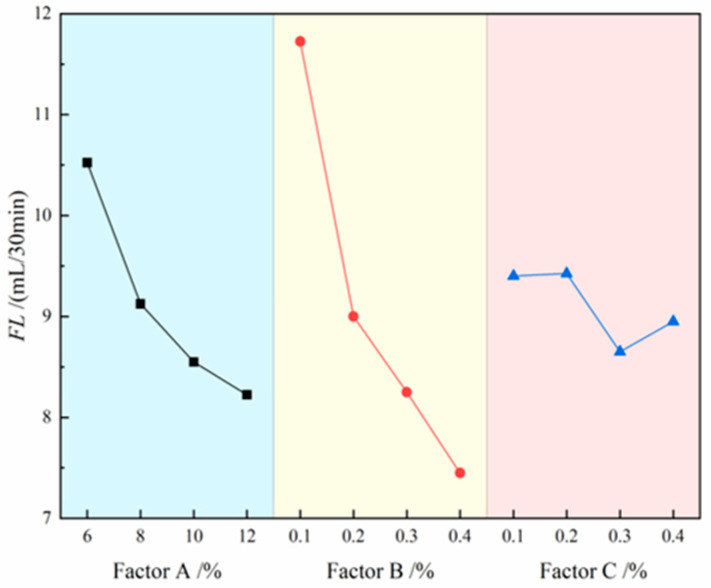
Effects of three factors on FL.

The contribution effects of three factors on FL are illustrated in Figure 7, where the four level values of each factor are represented on the *x*-axis and the FL values are indicated on the *y*-axis. The analysis results demonstrate that the degree of influence of each factor on slurry FL follows the following order: CMC > bentonite > Na_2_CO_3_. Specifically, FL shows a significant decreasing trend with increasing CMC and bentonite content, while it initially decreases and then increases with rising Na_2_CO_3_ content. The range analysis in Table 6 further confirms this conclusion, with the range values ranking as follows: CMC (R = 4.275) > bentonite (R = 2.3) > Na_2_CO_3_ (R = 0.775). Notably, FL exhibits a negative correlation with slurry performance, meaning that lower FL corresponds to better slurry performance. Therefore, when optimizing the slurry formulation, priority should be given to increasing the dosage of CMC and bentonite, while the Na_2_CO_3_ content needs to be controlled within an optimal range to achieve the best slurry performance. Ultimately, under the dual criteria of maintaining FL below 25 mL/30 min and prioritizing minimal values, the optimal combination for minimizing slurry fluid loss was determined to be A_4_B_4_C_3_.

**Figure 8:**
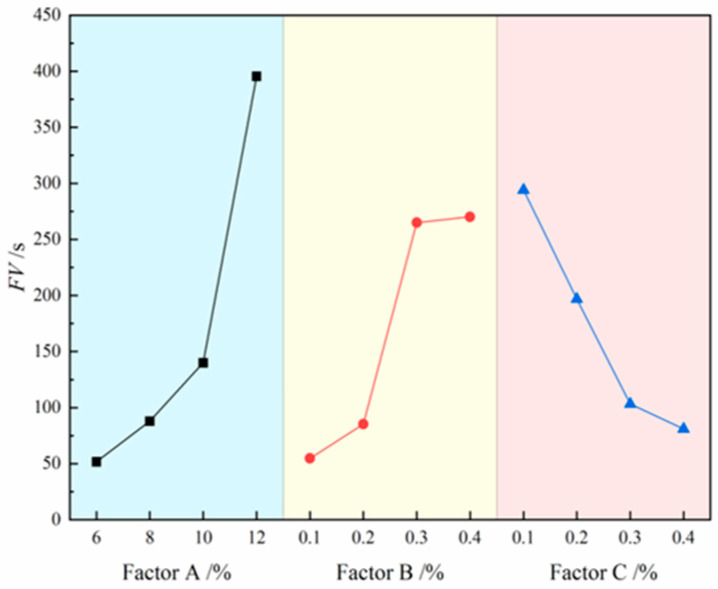
Effects of three factors on FV.

The contribution effects of three factors on FV are illustrated in Figure 8, where the four level values of each factor are represented on the *x*-axis and the FV values are indicated on the *y*-axis. The analysis results reveal that the degree of influence of each factor on slurry FV follows the following order: bentonite > CMC > Na_2_CO_3_. Specifically, FV shows a significant increasing trend with rising bentonite and CMC content, while it exhibits a clear decreasing trend with increasing Na_2_CO_3_ content. The range analysis in Table 7 further confirms this conclusion, with the range values ranking as follows: bentonite (R = 343.75) > CMC (R = 215.5) > Na_2_CO_3_ (R = 213). Notably, bentonite demonstrates the most pronounced effect on FV enhancement, as evidenced by its significantly higher range value compared to other factors, indicating its dominant role in regulating slurry viscosity. Although CMC and Na_2_CO_3_ show relatively smaller effects, their influence remains non-negligible. Ultimately, the A_3_B_2_C_3_ formulation was identified as the optimal composition for maintaining slurry FV within the target range of 100–120 s, achieving balanced rheological properties and stability.

**Figure 9:**
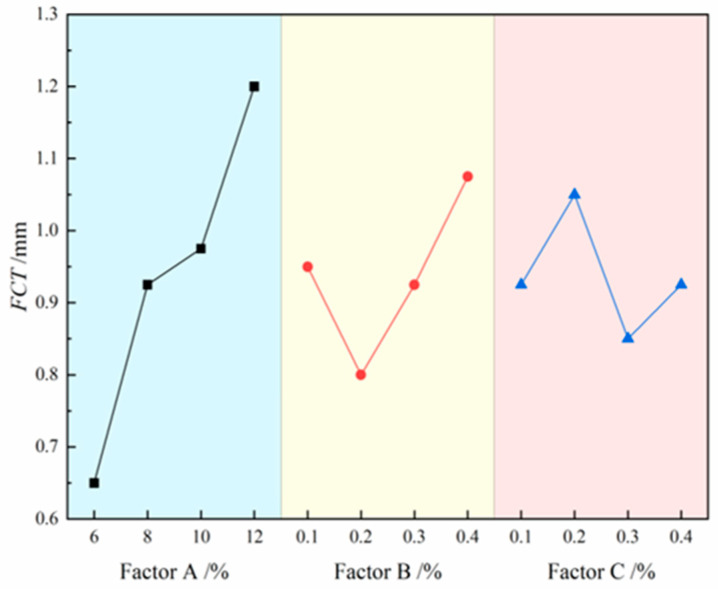
Effects of three factors on FCT.

The contribution effects of three factors on FCT are illustrated in Figure 9, where the four level values of each factor are represented on the *x*-axis and the FCT values are indicated on the *y*-axis. The analysis results demonstrate that the degree of influence of each factor on FCT follows the following order: bentonite > CMC > Na_2_CO_3_. Specifically, FCT shows a significant increasing trend with rising bentonite content, while it exhibits a fluctuating pattern of initial decrease followed by increase with increasing CMC content. For Na_2_CO_3_, the FCT displays a more complex trend of increase, decrease, and subsequent increase. The range analysis in Table 8 further confirms this conclusion, with the range values ranking as follows: bentonite (R = 0.55) > CMC (R = 0.275) > Na_2_CO_3_ (R = 0.2). The range values indicate that bentonite has the most pronounced effect on FCT, with its range value approximately twice that of CMC and 2.75 times that of Na_2_CO_3_, highlighting its dominant role in regulating FCT. Although both CMC and Na_2_CO_3_ exhibit relatively minor overall effects, their complex variation trends suggest potentially significant impacts on FCT within specific concentration ranges. Ultimately, through comprehensive analysis of optimal FL and FV combinations under the constraint of maintaining FCT within the 0.5–1 mm range, the A_3_B_2_C_3_ formulation was identified as achieving this target thickness criterion, despite multiple formulations meeting the basic FCT engineering requirements.

**Figure 10:**
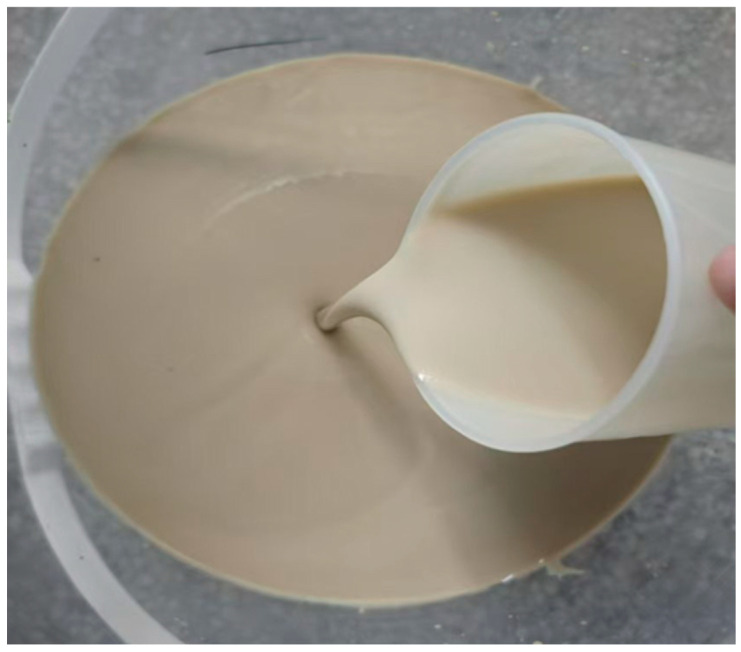
Photograph of the prepared optimal thixotropic slurry (A_3_B_4_C_3_).

Based on orthogonal experimental results, the A_3_B_3_C_4_ formulation (10 wt.% bentonite, 0.3 wt.% CMC, and 0.4 wt.% Na_2_CO_3_) demonstrated compliance with all six performance criteria within the specified control ranges (pH 11.09 ≈ 11) and exhibited superior overall performance compared to the other 15 tested formulations, thereby establishing it as the optimal slurry formulation. Figure 10 shows the prepared slurry under this formulation. Notably, range analysis revealed that none of the three factors exhibited significant effects on either slurry SG or pH, and all 16 experimental groups achieved zero WSR, demonstrating that all formulations met the fundamental stability requirements. Consequently, these parameters were not subjected to further detailed analysis in this study.

### 3.2. Analysis of Comparative Experimental Results

#### 3.2.1. Effect of HEC Content on Slurry Performance

According to the experimental data on the performance of thixotropic slurry with varying HEC dosages in Table 9, the incorporation of HEC exerts a significant regulatory effect on the rheological properties and stability of the system. The experimental results reveal that as the HEC dosage increases from 0.2% to 1%, the slurry SG exhibits a linear growth from 1.035 to 1.041, while the FV simultaneously rises from 43 s to 121 s, demonstrating a significant positive correlation. Notably, within this dosage range, the WSR of the slurry remains consistently at 0%, and the FCT is maintained within the ideal range of 0.8 to 1 mm, indicating excellent suspension stability and dense film-forming capability. Simultaneously, the evolution of the system’s pH value displays a non-linear characteristic of initially decreasing, then increasing, and finally decreasing again, which is primarily attributed to the dynamic influence of polar groups in HEC molecules on the double-electric layer structure of the slurry colloid.

Regarding key performance parameters, the FL exhibits an exponential decline with increasing HEC dosage, significantly decreasing from 15.5 mL in the 0.2% dosage group to 9.5 mL in the 1% dosage group (a reduction of 38.7%). This is closely related to the molecular structure characteristics of HEC. As a non-ionic water-soluble cellulose ether, the numerous hydroxyl (−OH) and hydroxyethyl (−CH_2_CH_2_OH) groups in its molecular chains form a three-dimensional hydration network through hydrogen bonding: (i) Hydrophilic groups strongly associate with water molecules, significantly enhancing the water-retention capacity of the system; (ii) Long-chain molecules entangle to create spatial steric hindrance, effectively inhibiting the sedimentation and separation of solid particles; (iii) The formation of hydration films effectively seals filtration channels, reducing permeability. These multiple mechanisms synergistically improve the fluid loss control performance of the slurry.

Experimental analysis confirms that HEC significantly enhances the rheological properties and stability of the slurry through its unique molecular structure and hydration mechanisms. Its thickening effect, fluid loss reduction characteristics, and colloidal stability advantages provide potential applications for thixotropic slurries in complex engineering environments.

#### 3.2.2. Effect of CMC Content on Slurry Performance

According to the experimental data on performance parameters of thixotropic slurry with varying CMC dosages in Table 10, as the CMC dosage increases from 0.2% to 1%, the SG of slurry rises from 1.036 to 1.041, and the FV significantly increases from 45 s to 112 s. This phenomenon indicates that CMC effectively enhances the structural strength of the slurry through molecular chain entanglement, thereby significantly improving the rheological properties of the slurry. The increase in FV further validates the thickening effect of CMC in the thixotropic slurry, providing essential assurance for the stability and fluidity of the slurry during construction.

Regarding the key performance parameters, the FL shows a clear negative correlation with increasing CMC dosage, decreasing from 14.6 mL at a 0.2% dosage to 9.2 mL at a 1% dosage (a reduction of 37.0%). This result confirms the adsorption and stabilization effects of the hydroxyl and carboxyl groups in CMC molecules on bentonite particles, significantly reducing the fluid loss of the slurry. Additionally, the pH value gradually decreases from 10.43 to 10.35, indicating that as an anionic cellulose ether, CMC dosage has a certain influence on the pH value of the thixotropic slurry. Notably, under all formulations, the WSR remains stable at 0%, and the FCT stays consistent at 1 mm. This demonstrates that the three-dimensional network structure formed by CMC and bentonite particles exhibits excellent anti-segregation stability and interfacial sealing properties. This stable structure not only effectively prevents stratification and water separation in the slurry but also provides reliable support for the long-term stability of the slurry during construction.

The findings highlight that CMC, as an additive for thixotropic slurry, demonstrates significant advantages in thickening, reducing fluid loss, and colloid stability. The three-dimensional network structure formed by CMC and bentonite particles not only enhances the structural strength of the slurry but also improves its rheological properties and construction stability. This conclusion further confirms the widespread applicability of CMC in thixotropic slurries.

#### 3.2.3. Comparison of the Effects of HEC and CMC on Slurry Performance

Graphs were plotted to show the variations in FV, FL, pH, and SG of the slurry with HEC and CMC contents. Polynomial fitting was applied to these curves to visualize the influence trends of both admixtures on slurry performance indicators. The comparative curves and analysis between HEC and CMC are summarized below. All fitted curves achieved R^2^ values greater than 0.9, indicating good model fitting.

**Figure 11:**
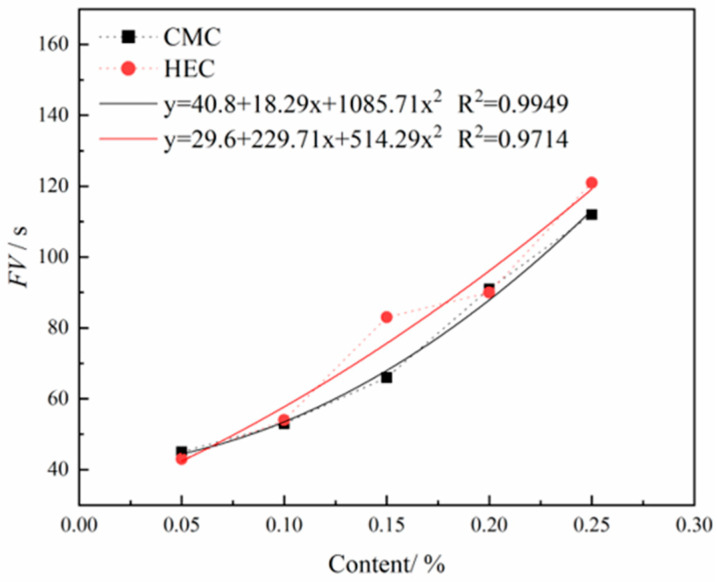
Effects of HEC/CMC content on FV.

As shown in Figure 11, the effects of HEC and CMC content on the FV of thixotropic slurry reveal the following trends: both cellulose derivatives exhibit a significant positive correlation between concentration and FV in their thickening effects. At dosages of 2–4% and 8%, there is no notable difference in FV between HEC and CMC. However, within the dosage ranges of 4–8% and 8–10%, HEC demonstrates a more pronounced enhancement in FV compared to CMC, indicating that HEC possesses a more significant thickening capability within certain dosage ranges.

**Figure 12:**
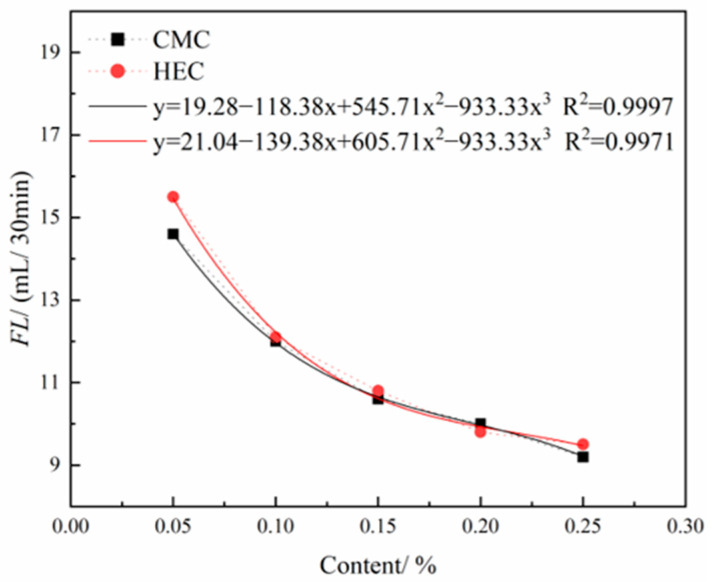
Effects of HEC/CMC content on FL.

As shown in Figure 12, the FL of the thixotropic slurry system shows a significant downward trend as the content of both cellulose-based admixtures gradually increases. The improvement effects of the two cellulose derivatives on the water-retention performance of the slurry are similar. Analysis indicates that this stems from the three-dimensional network structure formed by the polymer segments during hydration, which effectively blocks the microporous channels in the slurry system and inhibits the exudation of free water. Furthermore, curve fitting indicates that the FL of slurries containing either CMC or HEC additives both exhibit a negative correlation with additive content. Initially, the FL of the HEC-containing slurry is slightly higher than that of the CMC-containing slurry, but later becomes generally comparable. This phenomenon may be attributed to the increased viscosity of the system, which reduces the uniformity of dispersion. Therefore, in engineering practice, either CMC or HEC can be flexibly selected as water-retention enhancers based on material supply conditions and economic indicators.

**Figure 13:**
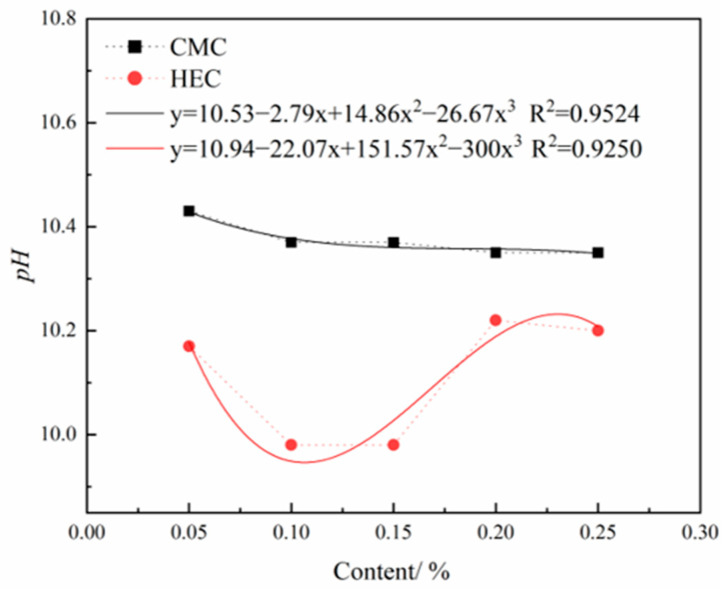
Effects of HEC/CMC content on pH.

As shown in Figure 13, under the same dosage conditions, the pH value of the slurry system incorporating CMC is significantly higher than that incorporating HEC. With increasing CMC dosage, the slurry pH exhibits a downward trend, while changes in HEC dosage result in a nonlinear pH variation, characterized by an initial decrease, followed by an increase, and then another decrease. Notably, throughout the entire dosage range, the pH value of the HEC slurry system remains consistently lower than that of the CMC slurry system. This discrepancy in mechanisms primarily stems from the differences in charge response characteristics in solution between non-ionic HEC and anionic CMC.

**Figure 14:**
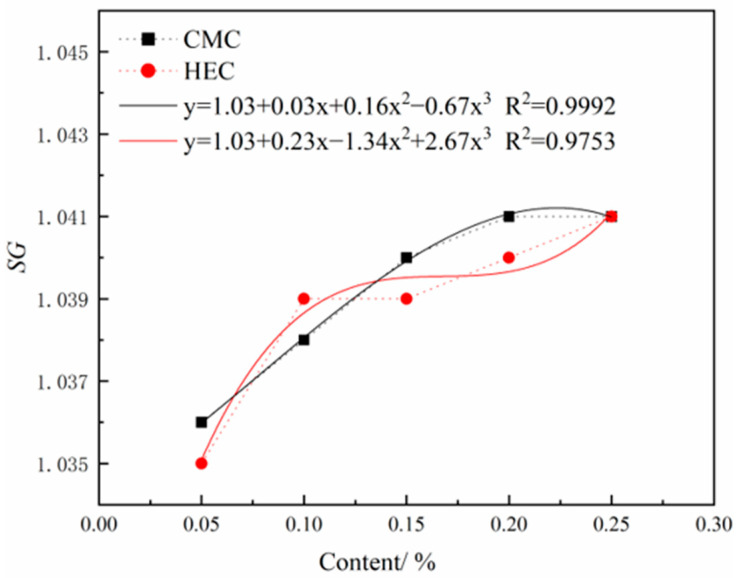
Effects of HEC/CMC content on SG.

As shown in Figure 14, the incorporation of both cellulose-based admixtures exerts a certain influence on the SG regulation of the slurry system, manifested by an increase in slurry SG with the rising dosage of the admixture. This phenomenon of SG enhancement is closely related to the molecular structures of the cellulose derivatives: the hydroxyethyl groups (−CH_2_CH_2_OH) in HEC molecules and the carboxymethyl groups (−CH_2_COO^−^) in CMC molecules can form stable associations with water molecules through hydrogen bonding. Meanwhile, their long-chain molecules generate steric hindrance effects in the slurry, significantly enhancing the suspension stability of solid particles, thereby inhibiting slurry sedimentation and improving overall compactness, ultimately leading to an increase in SG.

In conclusion, HEC and CMC exhibit similar rheological regulation characteristics in thixotropic slurries, but there are certain differences between the two in terms of thickening efficiency, FL control, pH influence, and SG regulation: HEC slightly outperforms CMC in thickening efficiency, while the two show significant differences in their impact on pH; in terms of FL control and SG enhancement, the effects of the two are comparable, effectively improving the construction performance and stability of the slurry. These findings provide an important basis for the selection of cellulose-based admixtures in engineering practice and also lay the foundation for the potential application of HEC in thixotropic slurries for pipe jacking construction.

### 3.3. Friction Test Results and Analysis

Based on orthogonal experiments and comparative experiments, five representative formulation combinations were selected, including orthogonal experiment groups A_3_B_4_C_3_ (FV 225 s), A_3_B_3_C_4_ (FV 119 s), A_1_B_1_C_1_ (FV 36 s), as well as comparative experiment groups CMC-0.25 (FV 112 s) and HEC-0.25 (FV 121 s).

**Figure 15:**
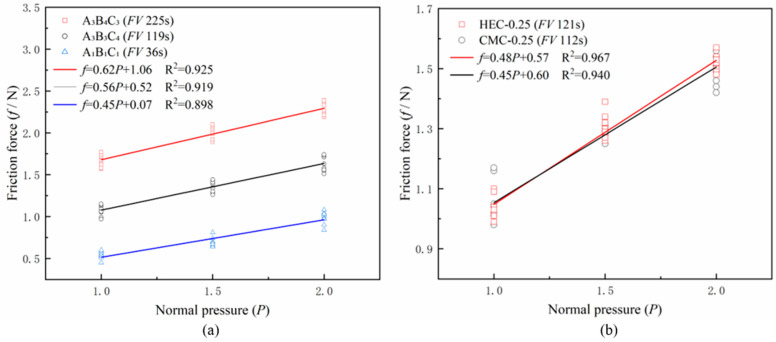
Scatter plots of friction characteristics with linear regression: (**a**) orthogonal experimental formulations, (**b**) comparative experimental formulations.

As shown in Figure 15a, the frictional characteristic tests of different viscosity formulations revealed that with increasing slurry viscosity, both the slope (friction coefficient) and intercept (adhesion force) of the fitted linear curve showed an upward trend. Specifically, the friction coefficient demonstrated an increasing progression of 0.45 → 0.56 → 0.62, while the adhesion force increased from 0.07 N to 1.06 N, which aligns with the theoretical expectation that viscosity affects interfacial friction characteristics. Further experimental observations indicated that while high-viscosity slurries (>200 s) could provide excellent support, they led to increased frictional resistance and reduced friction-reduction efficiency; whereas low-viscosity slurries (<50 s) showed significant friction-reduction effects but caused test block settlement due to insufficient support capacity. Therefore, slurry formulation optimization requires balancing friction-reduction performance and support capacity—maintaining medium viscosity (100–120 s) can achieve effective friction reduction while preserving stable support for pipe jacking. Within this optimal range, the A_3_B_3_C_4_ formulation (FV 119 s) demonstrated the best comprehensive performance.

As shown in Figure 15b, when bentonite content was fixed at 8%, CMC-0.25 (FV 112 s) and HEC-0.25 (FV 121 s) showed no significant differences in either friction coefficient (0.48 vs. 0.45) or adhesion force (0.57 N vs. 0.60 N), which also conformed to theoretical expectations.

### 3.4. Microstructure Analysis

**Figure 16:**
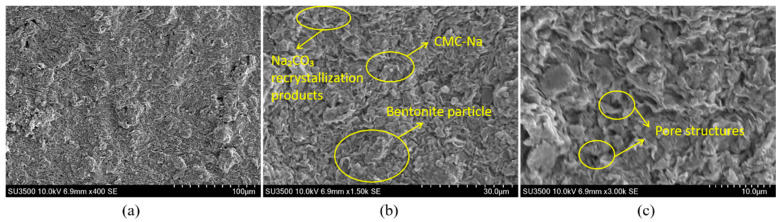
SEM images of the bentonite-CMC-Na_2_CO_3_ ternary system at different magnifications: (**a**) 400×, (**b**) 1500×, (**c**) 3000×.

Figure 16 illustrates the microstructural morphology of the thixotropic slurry in the bentonite-CMC-Na_2_CO_3_ ternary system. Under 400× magnification (Figure 16a), the sample exhibits a typical montmorillonite lamellar stacking structure, consistent with the morphological characteristics of layered silicate minerals. When magnified to 1500× (Figure 16b), the edges of the montmorillonite lamellae become more distinct. CMC molecules are uniformly coated on the bentonite surface via electrostatic adsorption and hydrogen bonding, while micron-sized crystalline particles, identified as Na_2_CO_3_ recrystallization products, are observed at partial pores and lamellar edges. Further magnification to 3000× (Figure 16c) reveals clearly distinguishable monolayer montmorillonite structures. CMC forms a continuous wrapping network through molecular chain crosslinking, constructing a uniform organic–inorganic composite interface on the bentonite surface. The regularly distributed pore structures provide effective channels for moisture migration.

**Figure 17:**
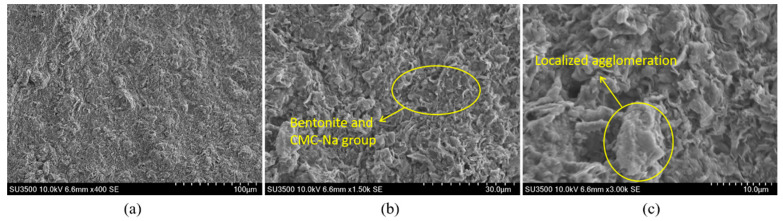
SEM images of the bentonite-CMC binary system at different magnifications: (**a**) 400×, (**b**) 1500×, (**c**) 3000×.

For comparative analysis, Figure 17 shows the microstructural features of the bentonite-CMC binary system for comparison. Under 400× and 1500× magnifications (Figure 17a,b), its macroscopic structure remains largely consistent with that of the Na_2_CO_3_-containing system. However, under 3000× high-resolution imaging (Figure 17c), localized agglomeration phenomena become apparent, accompanied by loosely stacked bentonite lamellae and uneven pore size distribution. This phenomenon can be attributed to the absence of two key mechanisms: (i) insufficient montmorillonite lamellar dispersion due to the lack of ion exchange induced by soda ash, and (ii) disordered entanglement of CMC molecular chains caused by charge imbalance. Such microstructural defects may lead to reduced water-retention capacity and impaired thixotropic performance.

**Figure 18:**
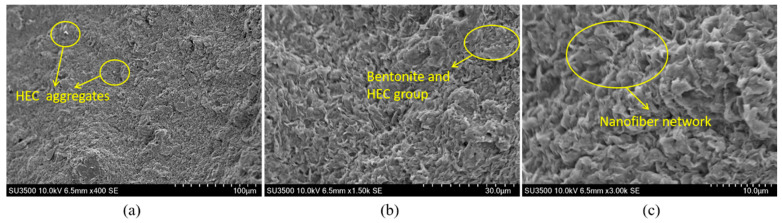
SEM images of the bentonite-HEC binary system at different magnifications: (**a**) 400×, (**b**) 1500×, (**c**) 3000×.

Figure 18 presents the microstructural morphology of the bentonite-HEC binary thixotropic slurry. Under 400× magnification (Figure 18a), bentonite retains its typical lamellar stacking morphology, while HEC molecular chains form localized bright white aggregates due to incomplete dispersion. Under 1500× magnification (Figure 18b), partial exfoliation of bentonite lamellae is observed, with HEC forming a thin film coating on the bentonite surface via intermolecular hydrogen bonding, exhibiting similarities to the CMC systems in Figure 13 and Figure 14. Further magnification to 3000× (Figure 18c) reveals three critical structural features: (i) HEC forming a continuous nanofiber network uniformly coating the bentonite surface and extending into interlayer spaces; and (ii) uniform overall pore distribution interspersed with localized fiber fractures and agglomerations. This composite structure endows the material with pronounced thixotropic properties through synergistic hydrogen-bonded networks and physical entanglement.

Further analysis indicates that CMC (existing as CMC-Na), an anionic cellulose ether, adsorbs onto bentonite particles via electrostatic interactions and hydrogen bonding to form a three-dimensional network, enhancing interparticle cohesion and improving slurry thixotropy [43]. In contrast, HEC, a non-ionic cellulose ether, establishes hydrogen bonds between its hydroxyl groups and surface hydroxyls or interlayer cations of bentonite lamellae, strengthening particle adhesion. These distinct properties enable targeted applications of the two cellulose ethers in different engineering scenarios. Additionally, the incorporation of appropriate soda ash facilitates uniform bentonite dispersion and promotes the formation of a more continuous three-dimensional network. This not only enhances slurry stability but also effectively retards the sedimentation rate of solid particles.

## 4. Conclusions

This study thoroughly investigates the influence mechanisms of bentonite, CMC, and Na_2_CO_3_ on the key performance parameters of thixotropic slurry through systematic orthogonal experiments and comparative experiments, and for the first time evaluates the potential application of HEC as a new additive in slurry for pipe jacking construction. Based on the experimental results and analysis, the following conclusions are drawn:

(1) Utilizing an orthogonal experimental design, this study quantified the effects of bentonite, CMC, and Na_2_CO_3_ concentrations on thixotropic slurry metrics (SG, pH, FL, FCT, WSR, FV). Range analysis revealed their influence hierarchy and identified the optimal formulation: 10 wt% bentonite, 0.3 wt% CMC, 0.4 wt% Na_2_CO_3_ (A_3_B_3_C_4_).
(2) Comparative experimental studies showed HEC regulates slurry rheology similarly to CMC, enhancing FV, reducing FL, and controlling WSR/FCT. Crucially, HEC yields significantly lower pH than CMC at equivalent dosages, indicating its advantage in high-salinity/acidic geologies.
(3) The slurry viscosity influences both the drag reduction efficiency (directly affecting pipe jacking performance) and the formation support capability, demonstrating a critical trade-off relationship in practical applications.
(4) This study has compared the performance differences between CMC and HEC when applied in thixotropic slurries, though it has not yet included a systematic statistical investigation of how HEC content affects various performance indicators of thixotropic slurries, nor has it verified the practical engineering effectiveness in highly saline formations. Given HEC’s promising application potential in pipe jacking thixotropic slurries and its current early-stage research status with insufficient technological maturity, future research should focus on the following: (i) Establishing synergistic systems combining HEC with sodium carbonate or other additives [44]; (ii) Establishing statistical relationship models between HEC content and slurry performance indicators through extensive experimental data to provide theoretical support for engineering applications; and (iii) Developing optimized HEC-based thixotropic slurry formulations tailored for challenging geological conditions.

## Figures and Tables

**Table 1:** Specifications of raw materials.

Name	Specification
Sodium bentonite	Montmorillonite content ≥ 75%
	Gel yield (swelling capacity): 100.2 mL/15 g
Na_2_CO_3_	Manufacturer: Shanghai Xilong Scientific Co., Ltd. (Shanghai, China)
	Purity ≥ 99.8% (chemically pure grade)
HEC	Viscosity of 2% aqueous solution: 5000–6000 mPa·s at 20 °C
CMC	Viscosity of 20 g/L solution: 800–1200 mPa·s at 25 °C
Water	Municipal tap water

**Table 2:** Parameter control requirements and instruments.

Parameter	Unit	Control Requirement	Experimental Instrument
SG	-	1.05–1.16	NB-1 Mud Balance
FV	s	100–120	Marsh Funnel
pH	-	8–11	PHB-4 Portable pH Meter
FL	mL/30 min	≤25	ZNA-2A Filter Press
FCT	mm	0.5–1	Caliper
WSR	%	0	1000 mL Graduated Cylinder

**Table 3:** Factors and levels of the orthogonal experiment.

No.	Factors	Level 1	Level 2	Level 3	Level 4
A	Bentonite content (%)	6.0	8.0	10.0	12.0
B	CMC content (%)	0.1	0.2	0.3	0.4
C	Na_2_CO_3_ content (%)	0.1	0.2	0.3	0.4

**Table 4:** Slurry formulations in the comparative experiment.

No.	Bentonite Content (%)	HEC Content (%)	CMC Content (%)	Water Content (%)
HEC-0.05	8	0.05	-	91.95
HEC-0.10	8	0.10	-	91.90
HEC-0.15	8	0.15	-	91.85
HEC-0.20	8	0.20	-	91.80
HEC-0.25	8	0.25	-	91.75
CMC-0.05	8	-	0.05	91.95
CMC-0.10	8	-	0.10	91.90
CMC-0.15	8	-	0.15	91.85
CMC-0.20	8	-	0.20	91.80
CMC-0.25	8	-	0.25	91.75

**Table 5:** Results of the orthogonal experiment.

No.	A (%)	B (%)	C (%)	SG	pH	FV (s)	WSR (%)	FL (mL/30 min)	FCT (mm)
1	6	0.1	0.1	1.020	10.68	36	0	13.7	0.7
2	6	0.2	0.2	1.023	10.80	43	0	11.0	0.5
3	6	0.3	0.3	1.030	10.98	56	0	9.0	0.6
4	6	0.4	0.4	1.031	11.11	72	0	8.4	0.8
5	8	0.4	0.1	1.038	10.70	183	0	8.0	1.0
6	8	0.3	0.2	1.040	10.89	82	0	8.9	1.1
7	8	0.2	0.3	1.038	11.02	49	0	8.2	0.7
8	8	0.1	0.4	1.037	11.11	38	0	11.4	0.9
9	10	0.2	0.1	1.053	10.57	154	0	8.3	0.9
10	10	0.1	0.2	1.050	10.77	62	0	11.4	1.1
11	10	0.4	0.3	1.051	10.93	225	0	7.0	1.0
12	10	0.3	0.4	1.058	11.09	119	0	7.5	0.9
13	12	0.3	0.1	1.068	10.68	803	0	7.6	1.1
14	12	0.4	0.2	1.070	10.80	601	0	6.4	1.5
15	12	0.1	0.3	1.069	10.96	83	0	10.4	1.1
16	12	0.2	0.4	1.068	11.11	95	0	8.5	1.1

**Table 6:** Results of range analysis for FL.

Factors	Mean FL Values (K_1_) Across Experimental Levels (mL/30 min)						
	Level 1	Level 2	Level 3	Level 4	R	Priority Order	Optimal Combination
A	10.525	9.125	8.550	8.225	2.300	B-A-C	A_4_B_4_C_3_
B	11.725	9.000	8.250	7.450	4.275		
C	9.400	9.425	8.650	8.950	0.775		

**Table 7:** Results of range analysis for FV.

Factors	Mean FV Values (K_2_) Across Experimental Levels (s)						
	Level 1	Level 2	Level 3	Level 4	R	Priority Order	Optimal Combination
A	51.750	88.000	140.000	395.500	343.750	A-B-C	A_3_B_2_C_3_
B	54.750	85.250	265.000	270.250	215.500		
C	294.000	197.000	103.250	81.000	213.000		

**Table 8:** Results of range analysis for FCT.

Factors	Mean FCT Values (K_3_) Across Experimental Levels (mm)						
	Level 1	Level 2	Level 3	Level 4	R	Priority Order	Optimal Combination
A	0.650	0.925	0.975	1.200	0.550	A-B-C	A_3_B_2_C_3_
B	0.950	0.800	0.925	1.075	0.275		
C	0.925	1.050	0.850	0.925	0.200		

**Table 9:** Results of the comparative experiment (HEC).

No.	Key Performance Indicators of Thixotropic Slurry					
	SG	FV (s)	WSR (%)	FCT (mm)	FL (mL/30 min)	pH
HEC-0.05	1.035	43	0	1.0	15.5	10.17
HEC-0.10	1.039	54	0	1.0	12.1	9.98
HEC-0.15	1.039	83	0	1.0	10.8	9.98
HEC-0.20	1.040	90	0	0.8	9.8	10.22
HEC-0.25	1.041	121	0	1.0	9.5	10.20

**Table 10:** Results of the comparative experiment (CMC).

No.	Key Performance Indicators of Thixotropic Slurry					
	SG	FV (s)	WSR (%)	FCT (mm)	FL (mL/30 min)	pH
CMC-0.05	1.036	45	0	1.0	14.6	10.43
CMC-0.10	1.038	53	0	1.0	12.0	10.37
CMC-0.15	1.040	66	0	1.0	10.6	10.37
CMC-0.20	1.041	91	0	1.0	10.0	10.35
CMC-0.25	1.041	112	0	1.0	9.2	10.35

## Data Availability

The original contributions presented in this study are included in the article. Further inquiries can be directed to the corresponding author.

## References

[1] Kaushal V., Najafi M., Serajiantehrani R. (2020). Environmental Impacts of Conventional Open-Cut Pipeline Installation and Trenchless Technology Methods: State-of-the-Art Review. J. Pipeline Syst. Eng. Pract..

[2] Ma P., Shimada H., Huang S., Moses D.N., Zhao G., Ma B. (2023). Transition of the pipe jacking technology in Japan and investigation of its application status. Tunn. Undergr. Space Technol..

[3] Dai W., Xu H., Liu S., Zhou W. (2019). Case Study of Small Diameter and Long Distance Steel Pipe Jacking Construction Crossing River. China Water Wastewater.

[4] Chen J., Li Z., Xu P. (2013). Design and Application of Large Diameter GRP Jacking Pipe in Long Distance Pipe Jacking Construction. China Water Wastewater.

[5] Fei Z. (2012). Key Technologies for Long Distance Curvilinear Pipe Jacking. China Water Wastewater.

[6] Li C., Zhong Z., Liu X., Tu Y., He G. (2019). Numerical simulation for an estimation of the jacking force of ultra-long-distance pipe jacking with frictional property testing at the rock mass-pipe interface. Tunn. Undergr. Space Technol..

[7] Yang R., Zeng D., Cui X. (2024). Research on Key Techniques for Backward Construction of Large Diameter Pipe Jacking under a Lake. China Water Wastewater.

[8] Yang X., Liu Y., Yang C. (2018). Research on the Slurry for Long-Distance Large-Diameter Pipe Jacking in Expansive Soil. Adv. Civ. Eng..

[9] Zhang D., Liu B., Qin Y. (2016). Construction of a large-section long pedestrian underpass using pipe jacking in muddy silty clay: A case study. Tunn. Undergr. Space Technol..

[10] Zhou S., Wang Y., Huang X. (2009). Experimental study on the effect of injecting slurry inside a jacking pipe tunnel in silt stratum. Tunn. Undergr. Space Technol..

[11] O’Dwyer K.G., McCabe B.A., Sheil B.B. (2020). Interpretation of pipe-jacking and lubrication records for drives in silty soil. Undergr. Space.

[12] Zhong X., Zhou Z., Li D. (2011). Key measures for large diameter steel pipe jacking engineering in Xijiang water diversion project and their practical effects. Water Wastewater Eng..

[13] Cheng W.-C., Wang L., Xue Z.-F., Ni J.C., Rahman M.M., Arulrajah A. (2019). Lubrication performance of pipejacking in soft alluvial deposits. Tunn. Undergr. Space Technol..

[14] Wang L., He J., Fan C. (2018). Research and Application of Drag Reduction by Grouting the Pipe Jacking in Complex Conditions. Mod. Tunn. Technol..

[15] Luo Z., Zhang Y., Chen J., Ou X., Zhang X. (2024). Stress and deformation response of pipe jacking in upper-soft and lower-hard strata: A case study in Changsha. Eng. Fail. Anal..

[16] Pellet-Beaucour A.L., Kastner R. (2002). Experimental and analytical study of friction forces during microtunneling operations. Tunn. Undergr. Space Technol..

[17] Liu S., Zhang B., Zhang X., Fan D., Wang H., Yu M. (2022). Formulation optimization and performance analysis of the thixotropic slurry for large-section rectangular pipe jacking in anhydrous sand. Constr. Build. Mater..

[18] Namli M., Guler E. (2017). Effect of Bentonite Slurry Pressure on Interface Friction of Pipe Jacking. J. Pipeline Syst. Eng. Pract..

[19] Wang Z., Wang J., He L., Gu X. (2024). Experimental Investigation of the Lubricant Effect of Thixotropic Slurry on Pipe-Soil Interfacial Friction Characteristics. Buildings.

[20] Zeng C., Xiao A., Liu K., Ai H., Chen Z., Zhang P. (2022). Experimental Study on the Influence of Slurry Concentration and Standing Time on the Friction Characteristics of a Steel Pipe-Soil Interface. Appl. Sci..

[21] Wen K., Shimada H., Zeng W., Sasaoka T., Qian D. (2020). Frictional analysis of pipe-slurry-soil interaction and jacking force prediction of rectangular pipe jacking. Eur. J. Environ. Civ. Eng..

[22] Shou K., Yen J., Liu M. (2010). On the frictional property of lubricants and its impact on jacking force and soil-pipe interaction of pipe-jacking. Tunn. Undergr. Space Technol..

[23] Cui G., Zhang H., Ma C., Zhang X., Shao H. (2024). Experimental study on optimization of pipe jacking mud mixture ratio based on MICP technology. Sci. Rep..

[24] Liu J., Wang X., Cheng H., Fan H. (2023). Orthogonal Design and Microstructure Mechanism Analysis of Novel Bentonite Polymer Slurry in Pipe Jacking. Polymers.

[25] Huang X., Sun J., Lv K., Liu J., Shen H., Zhang F. (2018). Application of core-shell structural acrylic resin/nano-SiO2 composite in water based drilling fluid to plug shale pores. J. Nat. Gas Sci. Eng..

[26] Huang X., Lv K., Sun J., Lu Z., Bai Y., Shen H., Wang J. (2019). Enhancement of thermal stability of drilling fluid using laponite nanoparticles under extreme temperature conditions. Mater. Lett..

[27] Liu K., Du H., Zheng T., Liu H., Zhang M., Zhang R., Li H., Xie H., Zhang X., Ma M. (2021). Recent advances in cellulose and its derivatives for oilfield applications. Carbohydr. Polym..

[28] Brachaczek W. (2018). Influence of Cellulose Ethers on the Consistency, Water Retention and Adhesion of Renovating Plasters. IOP Conf. Ser. Mater. Sci. Eng..

[29] Wang P., Zhao G., Zhang G. (2017). Mechanism on Water Retention and Thickening of Cellulose Ethers in Fresh Mortars. J. Chin. Ceram. Soc..

[30] Hynninen V., Patrakka J., Nonappa (2021). Methylcellulose-Cellulose Nanocrystal Composites for Optomechanically Tunable Hydrogels and Fibers. Materials.

[31] Arca H.C., Mosquera-Giraldo L.I., Bi V., Xu D., Taylor L.S., Edgar K.J. (2018). Pharmaceutical Applications of Cellulose Ethers and Cellulose Ether Esters. Biomacromolecules.

[32] Li X., Deng Q., Wang S., Li Q., Zhao W., Lin B., Luo Y., Zhang X. (2021). Hydroxyethyl Cellulose As a Rheological Additive for Tuning the Extrusion Printability and Scaffold Properties. 3d Print. Addit. Manuf..

[33] Ouaer H., Gareche M. (2019). Hydroxyethyl cellulose as a rheology modifier for water-based drilling fluids formulated with Algerian bentonite. J. Braz. Soc. Mech. Sci. Eng..

[34] Feng K., Ma K., Yang H., Long G., Xie Y., Zeng X., Tang Z., Usman I.U. (2024). Influence of cellulose ethers on rheological properties of cementitious materials: A review. J. Build. Eng..

[35] Kokol V. (2022). Influence of hydroxyethyl and carboxymethyl celluloses on the rheology, water retention and surface tension of water-suspended microfibrillated cellulose. Cellulose.

[36] Pan Y., Wang J., Yang S., Fu J., Eteme Y.L. (2023). Research progress of hydroxyethyl cellulose materials in oil and gas drilling and production. Cellulose.

[37] Benyounes K., Remli S., Benmounah A. (2017). Rheological behavior of Hydroxyethylcellulose (HEC) Solutions. J. Phys. Conf. Ser..

[38] Torrijos R., Nazareth T.M., Calpe J., Quiles J.M., Manes J., Meca G. (2022). Antifungal activity of natamycin and development of an edible film based on hydroxyethylcellulose to avoid Penicillium spp. growth on low-moisture mozzarella cheese. Lwt-Food Sci. Technol..

[39] Gospodinova A., Nankov V., Tomov S., Redzheb M., Petrov P.D. (2021). Extrusion bioprinting of hydroxyethylcellulose-based bioink for cervical tumor model. Carbohydr. Polym..

[40] Turczyn R., Weiss P., Lapkowski M., Daculsi G. (2000). In situ self hardening bioactive composite for bone and dental surgery. J. Biomater. Sci.-Polym. Ed..

[41] Wang M., Liu D. (2016). Test of Thixotropic Slurry Properties and Study of Resistance-Reducing Technology for Pipe Jacking Tunnel Construction. Mod. Tunn. Technol..

[42] Yang X.L., Liu G., Li Y., Gao S.H. (2021). Structural Optimization of Reciprocating Seal with Magnetic Fluid Based on Orthogonal Test Design. J. Magn..

[43] Benchabane A., Bekkour K. (2006). Effects of anionic additives on the rheological behavior of aqueous calcium montmorillonite suspensions. Rheol. Acta.

[44] Li B., Shao Z., Liao B. (2010). Viscosity and Viscoelasticity Study on Association of HEC/CMC Mixed System in Aqueous Medium. Trans. Beijing Inst. Technol..

